# Therapeutic effect of ofatumumab in patients with myasthenia gravis: immunoregulation of follicular T helper cells and T helper type 17 cells

**DOI:** 10.3389/fneur.2023.1278250

**Published:** 2023-12-11

**Authors:** Shasha Li, Zhaoxu Zhang, Zunjing Liu

**Affiliations:** ^1^Graduate School of Beijing University of Chinese Medicine, Beijing, China; ^2^Department of Neurology, China-Japan Friendship Hospital, Beijing, China; ^3^Department of Neurology, Peking University People's Hospital, Beijing, China

**Keywords:** myasthenia gravis, ofatumumab, follicular T helper cells, T helper type 17 cells, B cells depletion

## Abstract

**Introduction:**

This study aimed to study the therapeutic effects of ofatumumab in patients with myasthenia gravis (MG) in addition to the immunomodulatory effects on peripheral follicular T helper (Tfh) cells and T helper type 17 (Th17) cells.

**Methods:**

Thirty-one patients with anti-acetylcholine receptor (AChR) antibody-positive MG were included in this study. At weeks 0, 1, 2, and 4, an initial dose of 20 mg of ofatumumab was injected subcutaneously, with a 2-month follow-up after completing this first cycle. At baseline, 1 month, and 3 months, we assessed the Quantitative MG (QMG), 15-item MG-Quality of Life (MG-QOL15), and MG-Activities of Daily Living (MG-ADL) scales and measured the frequencies of Tfh, Th17, and B cells and the levels of anti-AChR antibody, IL-6, IL-21, and IL-17 in the peripheral blood.

**Results:**

At 1 month and 3 months, the QMG, MG-QOL15, and MG-ADL scores were all significantly reduced. At 3 months, doses of prednisone were reduced by an average of 37%. Decreased frequencies of Tfh and Th17 cells, depletion of B cells, and reduced levels of IL-6, IL-21, and IL-17 were all observed at 1 month or 3 months.

**Discussion:**

Therefore, the therapeutic effect of ofatumumab could be detected after one cycle of treatment, which was maintained for 2 months. The immunomodulatory effect of ofatumumab during the observation period may involve depletion of B cells, reduction of Tfh and Th17 cells frequencies, and reduced levels of IL-6, IL-21, and IL-17. The findings provide novel data for the potential application of ofatumumab in MG.

## 1 Introduction

Myasthenia gravis (MG) is a chronic organ-specific autoimmune disease caused by autoantibodies directed against components of the neuromuscular junction. Acetylcholine receptor (AChR) autoantibodies are observed in approximately 85% of MG patients (1). MG is clinically characterized by partial or generalized muscle weakness and fatigue, aggravated with activity and relieved with rest (2). MG is a rare disease, with an estimated incidence of 0.3 to 2.8 per 100,000 people and a global prevalence of 700,000 (3). MG can affect daily activities, and if untreated, approximately 20% of patients will suffer exacerbated muscle weakness and respiratory failure.

Although MG is an antibody-mediated autoimmune disease, antibody synthesis requires the intervention of cellular immunity. The peripheral immune response, which is regulated by autoreactive T cells and B cells that have escaped central tolerance, is the primary pathogenesis of MG (4, 5). CD4+ T cells serve as the main driver in the immune pathogenesis of MG; the redistribution of CD4+ T cells leads to an increase in the frequency of follicular T helper (Tfh) cells and T helper type 17 (Th17) cells (6, 7). Circulating Tfh cells move between the peripheral blood and lymphoid tissue and are the most potent regulators of humoral immunity (8). Th17 cells contribute to the induction of tissue inflammation in autoimmune diseases (9). The increased frequency of Tfh cells and Th17 cells was reported to significantly exacerbate the development of MG in humans and experimental models (10–12). Autoreactive B cells, coordinated primarily by CD4+ T cells and cytokines, produce anti-AChR autoantibodies at the neuromuscular junction in MG, leading to the loss of AChR function at the postsynaptic membrane, impaired neuromuscular transmission, and clinical symptoms of muscle weakness (13). Therefore, similar to B cells, Tfh cells and Th17 cells also play an important role in the immune mechanism of MG.

At present, there is no consensus on the ideal therapeutic approach for MG. Targeted immunotherapy appears to be the most promising treatment avenue for MG, effectively overcoming the limitations of the currently applied non-specific immunotherapy and potentially inducing remission (14). Ofatumumab is a fully human second-generation anti-CD20 monoclonal antibody for subcutaneous application. It binds to the CD20 molecule on the B-cell surface and induces B-cell depletion through antibody-dependent and complement-dependent cytotoxicity (15). Ofatumumab has been approved for the treatment of relapsing multiple sclerosis (MS) in adults (16). Rituximab, another monoclonal antibody directed against CD20, provided a meaningful and prolonged benefit in >70% of anti-AChR antibody-positive MG patients (17). However, to the best of our knowledge, there is only one brief report regarding the effectiveness of ofatumumab in treating one patient with refractory MG (18).

To investigate the clinical efficacy as well as the immunomodulatory effects of ofatumumab in MG patients, we evaluated several clinical scales and detected the frequency of Tfh cells, Th17 cells, and B cells, in addition to the levels of anti-AChR antibodies and related cytokines in the peripheral blood. The findings will provide a novel idea for applying ofatumumab in MG.

## 2 Materials and methods

### 2.1 Patients

Subjects gave their written informed consent, and the research protocol was approved by the ethics committee of Peking University People's Hospital (2023PHB244-001) before the initiation of this study. The study was conducted in accordance with the World Medical Association Declaration of Helsinki.

**Figure 1:**
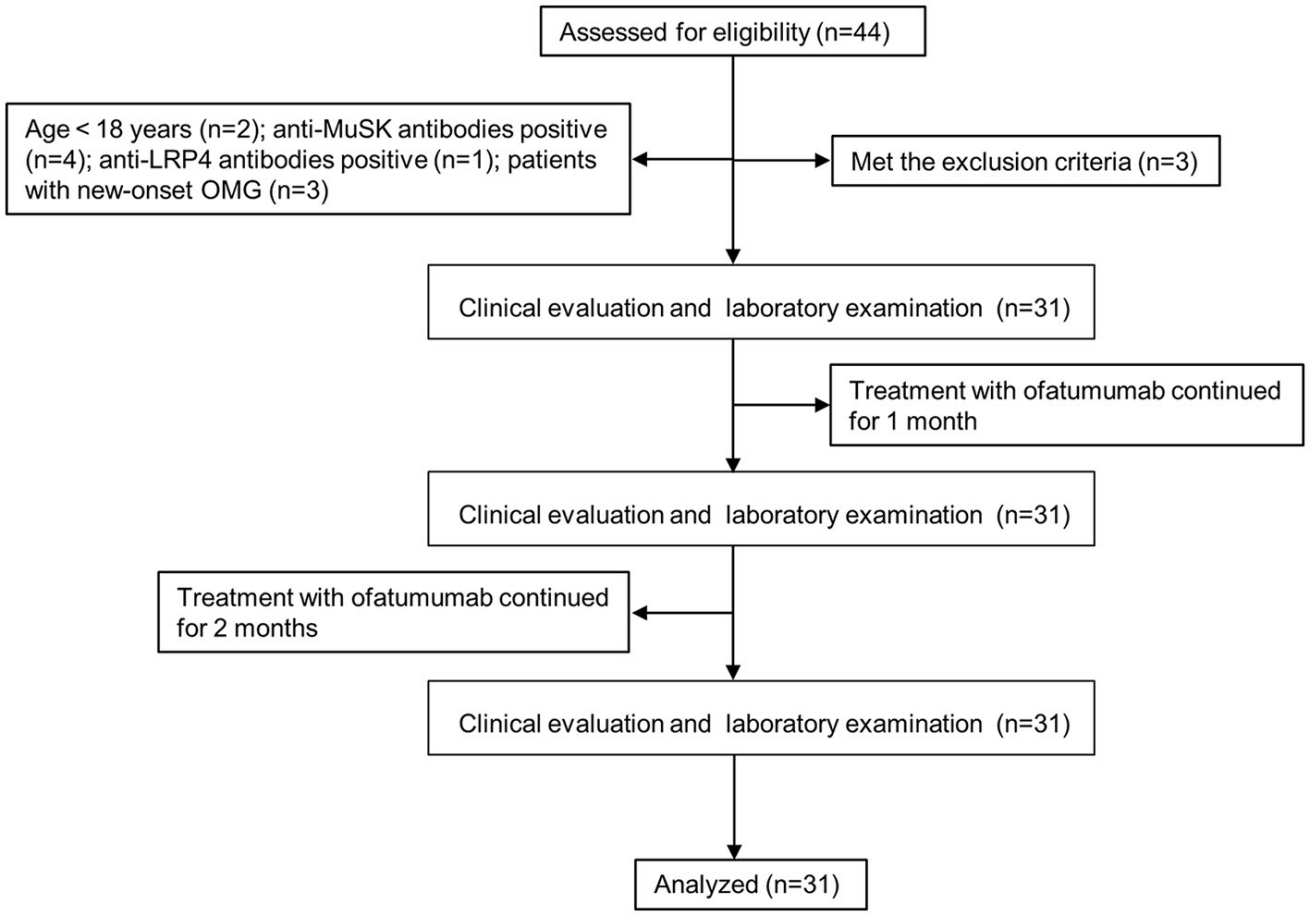
Flow diagram. OMG, ocular myasthenia gravis; MuSK, muscle-specific tyrosine kinase; LRP4, low-density lipoprotein receptor-related protein 4.

We collected data from cases who met the following criteria from January 2023 to April 2023 in the Department of Neurology at Peking University People's Hospital. The study flow diagram is shown in Figure 1. The inclusion criteria were as follows: (1) age ≥ 18 years; (2) diagnosis of MG based on the clinical evidence of muscle weakness and fatigue and at least one positive ancillary diagnostic test, including a positive response to the neostigmine test; (3) anti-AChR antibody-positive; (4) patients with Myasthenia Gravis Foundation of America (MGFA) clinical classification I~V (for patients with thymectomy or patients without thymoma, class I indicates weakness only in ocular muscles, class II for mild generalized disease, class III for moderate generalized disease, class IV for severe generalized disease, and class V for a crisis requiring intubation) (19); (5) class II~V patients, who did not respond to multiple immunosuppressive therapies, have intolerable adverse effects, require repeated intravenous immunoglobulin therapy or plasma exchange, have frequent MG crises (20), or have not received any immunosuppressive therapies; (6) class I patients who did not respond to multiple immunosuppressive therapies or have intolerable adverse effects; and (7) no prior treatment with ofatumumab. The exclusion criteria were as follows: (1) the co-presence of MG and malignant tumors, major organ damage, systemic hematological diseases, and recent infections; (2) pregnancy or lactation; and (3) patients who failed to give or comply with informed consent.

### 2.2 Ofatumumab and other therapies

Before starting ofatumumab treatment, patients were required to discontinue immunosuppressive agents in case of unexpected compromise to the immune system. Patients were allowed to continue taking oral prednisone, and the dose was adjusted according to clinical response during the treatment period. At weeks 0, 1, 2, and 4, an initial dose of 20 mg was injected subcutaneously. Periodic clinical and laboratory assessments were performed after the completion of the first cycle. Patients were permitted to receive a repeat cycle of 20 mg once a month according to assessment results and patient's preference.

### 2.3 Clinical evaluation

Disease activity was assessed using the Quantitative MG (QMG) scale of the MGFA (19), 15-item MG-Quality of Life (MG-QOL15) scale (21), and MG-Activities of Daily Living (MG-ADL) scale (22) before treatment and at 1 and 3 months after the initiation of ofatumumab treatment.

### 2.4 Flow cytometry

At 0, 1, and 3 months, heparinized peripheral blood from patients with MG was collected and processed within 24 h. Peripheral blood mononuclear cells (PBMCs) were isolated by Ficoll-Hypaque density gradient centrifugation. PBMCs were stimulated and incubated for 5 h with 50 ng/mL phorbol 12-myristate 13-acetate (PMA; Sigma-Aldrich, Steinheim, Germany) and 500 ng/mL ionomycin (Sigma-Aldrich, Steinheim, Germany) in the presence of 10 μg/mL Brefeldin-A (BFA; BD Biosciences, San Diego, California, USA). The PBMCs were then incubated with the following fluorochrome-conjugated anti-human murine monoclonal antibodies: FITC-CD4, Alexa Fluor 647-CXCR5, Brilliant Violet 510-CD45RA, PE-CF594-CD19, PE-CD16, and PE-CD56 for 30 min at 4°C in the dark to stain the cell surface markers. Next, the cells were fixed and permeabilized for 30 min at room temperature in the dark with the Foxp3 Fixation/Permeabilization Concentrate and Diluent Buffer Set (eBioscience, San Diego, California, USA). PBMCs were then incubated for 30 min in the dark at 4°C with PE-labeled IL-17A antibody for intracellular cytokine staining. All antibodies were purchased from BD Biosciences (Franklin Lakes, New Jersey, USA) and BioLegend (San Diego, California, USA). Tfh cells were defined as CD4+CXCR5+CD45RA-, Th17 cells were defined as CD4+ T cells secreting IL-17A, and B cells were defined as CD19+ and not expressing CD16 and 56. After proper instrument set up, measurements were performed using a FACS Aria II flow cytometer (BD Biosciences, San Jose, California, USA), and FlowJov10 (Tree Star, Woodburn, OR) was applied for data analysis.

### 2.5 Enzyme-linked immunosorbent assay

The serum was separated from the peripheral blood by centrifugation (3,000 rpm, 10 min). The serum levels of interleukin (IL)-6, IL-21, IL-17, and anti-AChR antibodies in patients with MG were measured using the corresponding ELISA kits (Cusabio, Wuhan, China). The diluted serum and standard were added to each well to bind with the specific antibody for incubation, followed by horseradish peroxidase-labeled streptavidin and chromogenic substrate, and when the sample turned blue, the stop solution was added. The optical density at the absorbance of 450 nm was measured.

### 2.6 Statistical analysis

The statistical analysis was conducted using SPSS 26.0 software (SPSS Inc., Chicago, IL, USA) and GraphPad Prism 5 software (GraphPad Software Inc., La Jolla, CA, USA). Continuous variables were presented as mean ± standard deviation (SD) and categorical data were presented as percentages. To determine whether the data used for comparison between groups followed the normal distribution, the Shapiro–Wilk test was performed. The Student's *t*-test was used to analyze normally distributed data and the non-parametric Wilcoxon test for non-normally distributed data. A *P*-value of < 0.05 (two-tailed) was considered to be statistically significant.

## 3 Results

### 3.1 Clinical characteristics

In total, 31 subjects were included in the study (17 females and 14 males). The mean subject age was 53.32 ± 14.56 years and the disease duration was 18.42 ± 15.09 months. Ten patients with thymoma had undergone thymectomy. Of the 31 patients included, 24 were patients with refractory MG and seven were patients with new-onset MG who had not received prior treatment for MG. These 24 patients discontinued all other immunosuppressive agents, except prednisone, before the use of ofatumumab. During the study period, all 31 patients received the first cycle of ofatumumab, followed by a 2-month follow-up. Doses of prednisone were reduced by an average of 37% at 3 months in 24 patients, although none were able to free from prednisone entirely. No patients developed MG crises during the study period (Table 1).

**Table 1:** Clinical characteristics of enrolled patients with myasthenia gravis (MG).

**Patient no**.	**Sex**	**Age (years)**	**Disease duration (months)**	**Thymoma**	**MGFA type**	**All previous therapies**	**Ofatumumab dosage/cycle**	**Other therapy changes except pred**	**Pred dose change (%)**
1	F	62	49	B1	IIIb	Pred, AZA, Tx	80 mg/1	Stopped	33
2	F	58	6	None	I	Pred, PB	80 mg/1	Stopped	58
3	M	45	5	None	I	Pred, PB	80 mg/1	Stopped	48
4	M	32	38	AB	IVb	Pred, IVIg, Tx	80 mg/1	Stopped	20
5	F	75	26	None	IIb	Pred, AZA	80 mg/1	Stopped	43
6	F	52	41	B2	IIb	Pred, CYC A, Tx	80 mg/1	Stopped	50
7	M	53	5	None	IIa	None	80 mg/1	None	None
8	M	66	26	None	I	Pred, PB	80 mg/1	Stopped	44
9	M	35	3	None	IIb	None	80 mg/1	None	None
10	F	67	14	B1	IVb	Pred, IVIg, CYC A, Tx	80 mg/1	Stopped	17
11	F	72	17	None	IIIb	Pred, AZA	80 mg/1	Stopped	38
12	F	63	15	None	IIIb	Pred, AZA, CYC A	80 mg/1	Stopped	29
13	M	66	28	None	IIIa	Pred, CYC A	80 mg/1	Stopped	40
14	F	48	6	None	IIb	None	80 mg/1	None	None
15	F	55	50	AB	V	Pred, IVIg, AZA, Tx	80 mg/1	Stopped	25
16	F	72	38	None	IIb	Pred, AZA	80 mg/1	Stopped	33
17	F	65	24	B2	IIIa	Pred, CYC A, Tx	80 mg/1	Stopped	50
18	M	27	5	None	IIa	None	80 mg/1	None	None
19	F	68	12	B1	IIIa	Pred, AZA, Tx	80 mg/1	Stopped	25
20	F	59	36	None	IIIa	Pred, AZA	80 mg/1	Stopped	30
21	F	52	3	None	IIa	None	80 mg/1	None	None
22	M	33	17	None	IVa	Pred, IVIg, AZA	80 mg/1	Stopped	21
23	M	67	2	None	IIb	None	80 mg/1	None	None
24	F	35	28	B1	IIa	Pred, CYC A, Tx	80 mg/1	Stopped	33
25	M	25	14	None	I	Pred, PB	80 mg/1	Stopped	50
26	F	52	38	B2	IIb	Pred, AZA, CYC A, Tx	80 mg/1	Stopped	40
27	M	44	6	None	I	Pred, PB	80 mg/1	Stopped	43
28	M	65	5	None	IIIa	None	80 mg/1	None	None
29	F	63	3	B2	IIIb	Pred, AZA, CYC A, Tx	80 mg/1	Stopped	50
30	M	42	5	None	IIb	Pred, AZA	80 mg/1	Stopped	33
31	M	35	6	None	IIb	Pred, AZA, CYC A	80 mg/1	Stopped	30

MGFA, Myasthenia Gravis Foundation of America; Pred, prednisolone; AZA, azathioprine; Tx, thymectomy; PB, pyridostigmine bromide; IVIg, intravenous immunoglobulin; CYC A, cyclosporine.

### 3.2 Clinical efficacy of ofatumumab

**Figure 2:**
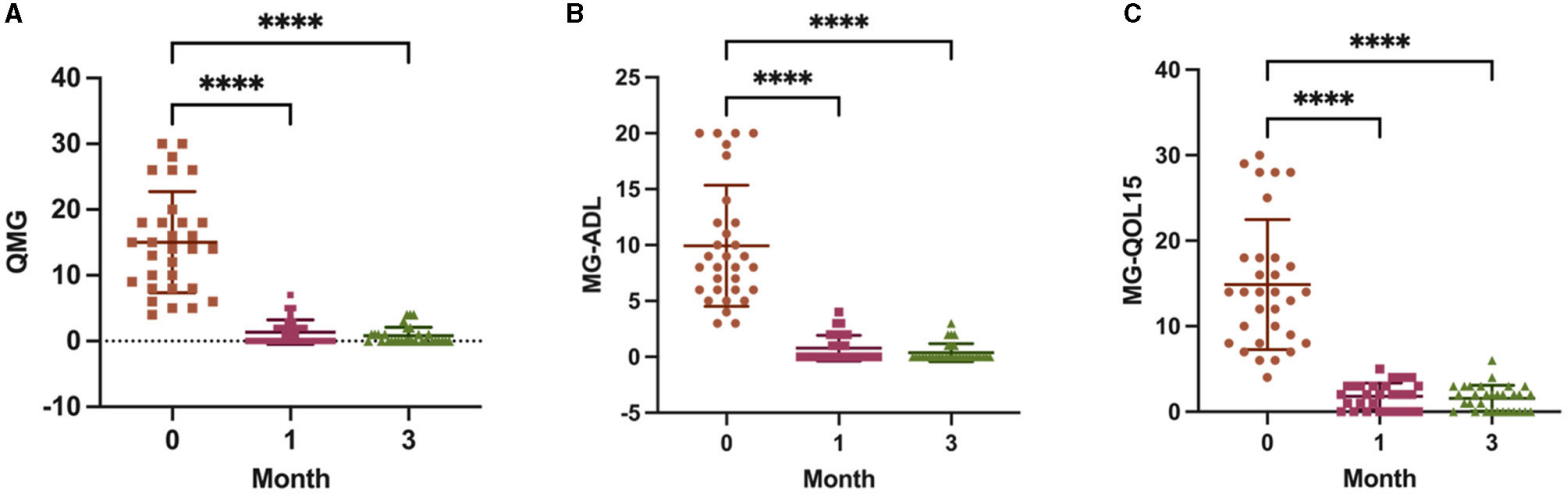
Scores from the Quantitative Myasthenia Gravis (QMG) **(A)**, 15-item MG-Quality of Life (MG-QOL15) **(B)**, and MG-Activities of Daily Living (MG-ADL) **(C)** assessments at baseline, 1 month, and 3 months. ****P < 0.0001 vs. baseline.

A significant decrease in the QMG, MG-QOL15, and MG-ADL scores was observed at 1 month of ofatumumab treatment (*P* < 0.0001), and the scores were maintained at 3 months (*P* < 0.0001) (Figure 2). In terms of safety, three patients (9.68%) reported mild adverse events during the study period, including one patient with mild fever and two patients with headaches, nasal congestion, and sore throat, all of which resolved on their own. The other patients tolerated the treatment well.

### 3.3 Ofatumumab reduced the Tfh and Th17 cells frequencies and depleted B cells

**Figure 3:**
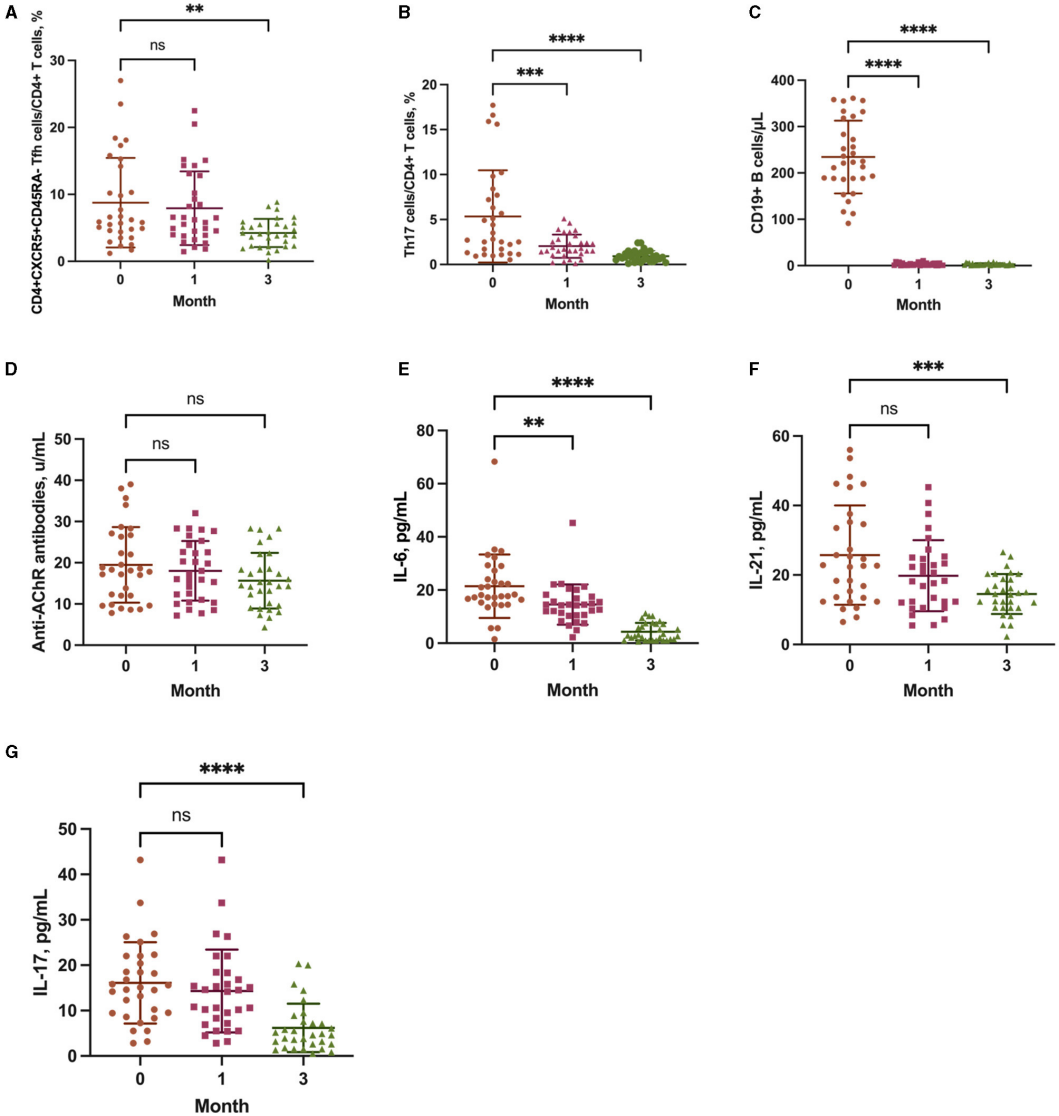
The frequency of CD4+CXCR5+CD45RA follicular T helper (Tfh) cells **(A)**, T helper type 17 (Th17) cells **(B)**, and CD19+ B cells **(C)**, and the levels of anti-AChR antibodies **(D)**, interleukin (IL)-6 **(E)**, IL-21 **(F)**, and IL-17 **(G)** at baseline, 1 month, and 3 months. **P < 0.01, ***P < 0.001, and ****P < 0.0001 vs. baseline.

The proportion of Tfh cells in CD4+ T cells was unchanged at 1 month of ofatumumab treatment (*P* = 0.8051) and significantly decreased at 3 months (*P* = 0.0024) (Figure 3A). However, the proportion of Th17 cells in CD4+ T cells was consecutively and significantly decreased at 1 (*P* = 0.0002) and 3 months (*P* < 0.0001) (Figure 3B). Furthermore, B-cell depletion was achieved at both 1 and 3 months (*P* < 0.0001) (Figure 3C). The anti-AChR antibody titer did not change significantly at 1 (*P* = 0.7476) and 3 months (*P* = 0.1350) (Figure 3D).

### 3.4 Ofatumumab decreased the levels of IL-6, IL-21, and IL-17

The levels of IL-6 (*P* = 0.0049), IL-21 (*P* = 0.0787), and IL-17 (*P* = 0.6512) were significantly decreased at 1 month of ofatumumab treatment and were consistently decreased at 3 months (IL-6, *P* < 0.0001; IL-21, *P* = 0.0002; IL-17, *P* < 0.0001) (Figures 3E–G).

## 4 Discussion

The effects of ofatumumab in patients with MG have yet to be explored. In this study, we found that ofatumumab has a therapeutic effect in patients with anti-AChR antibody-positive MG and that the immunomodulatory effect may involve the regulation of Tfh cells and Th17 cells. This study is the first to report the role of ofatumumab in MG, providing evidence for the therapeutic application of ofatumumab in the treatment of MG and a future direction to explore the therapeutic mechanism of ofatumumab in MG.

Only one case report has reported the effectiveness of ofatumumab in a patient with refractory MG after receiving two infusions of ofatumumab (700 mg) 2 weeks apart (18). This dose (700 mg) is relatively large; in the present study, we used a standard dose of 20 mg and followed up with the patients after completing the first cycle (20 mg at weeks 0, 1, 2, and 4). This dosage regimen was consistent with that reported in the recent review on the treatment of relapsing MS with ofatumumab (23). Similarly, the standard protocol of rituximab is 375 mg/m^2^ weekly for 4 weeks, after which investigators have used varying numbers of cycles with retreatments (17, 24, 25). In addition, many studies have suggested that repeated rituximab infusion, if clinically indicated, should be considered at 6 months after a cycle (17, 26). In the present study, clinical efficacy and B-cell depletion were maintained for 3 months after completing one cycle; however, the reason for this may be that our observation period was only 3 months.

The QMG, MG-QOL15, and MG-ADL are validated outcome measures that can be used to assess the therapeutic effect of interventions in MG clinical trials. QMG is a standardized and reliable scale to quantitatively assess a patient's clinical status and is the primary outcome indicator (27). MG-QOL15 is used to assess patients' health-related quality of life (28). MG-ADL correlates well with the QMG and can be used as a secondary measurement of clinical efficacy (22). In this study, the QMG, MG-QOL15, and MG-ADL scores of patients with MG were all significantly reduced after treatment with ofatumumab. Therefore, these results indicate that ofatumumab has a therapeutic effect in patients with MG; ofatumumab can reduce the severity of the disease and improve the quality of life. Rituximab has been used to treat MG for more than a decade and was thought to be effective in treating refractory MG patients; however, the improvement in the QMG, MG-ADL, or MG-QOL15 scores was often reported after 6 months of treatment (29, 30). Rituximab appeared to be a potentially effective treatment option for new-onset MG, which significantly improved the QMG, MG-ADL, and MG-QOL15 scores; a significant change in the QMG score was even detected after 1 month of treatment (31, 32). In contrast to rituximab, patients enrolled in this study included those with both refractory MG and new-onset MG, with significant reductions in the QMG, MG-ADL, and MG-QOL-15 scores at 1 month of treatment with ofatumumab, although this needs to be validated in a large-scale randomized controlled trial.

Tfh cells are a distinct subset of CD4+ T cells. In the immune response, Tfh cells are specialized helpers of B cells, including promoting the maturation of B cells and inducing the differentiation of B cells into antibody-producing plasma cells (PCs) and long-lived memory B cells. Tfh cells were hypothesized to be a target for preventing B cells from producing autoantibodies against their own antigens (33, 34). IL-21 is mainly secreted by Tfh cells and plays a crucial role in B cells proliferation, PCs differentiation, and antibody secretion (35, 36). A study reported that inhibition of Tfh cells transcription decreased the frequency of Tfh cells and IL-21 production (37). The Tfh cells frequency and IL-21 level were increased in patients with anti-AChR antibody-positive MG and were significantly correlated with antibody titer and disease severity (38, 39). In this study, treatment with ofatumumab significantly reduced the Tfh cells frequency and IL-21 level in patients with anti-AChR antibody-positive MG. These results indicate that the immunoregulatory effect of ofatumumab in MG might involve a reduction in the Tfh cells frequency and IL-21 level.

Th17 cells are highly pro-inflammatory and result in increased chronic inflammation at the MG neuromuscular junction through the release of cytokines, such as IL-17 (7). IL-17 has been shown to be involved in the immune response in various autoimmune diseases, such as MS, rheumatoid arthritis, systemic lupus erythematosus, and MG. The pro-inflammatory factor IL-17 is emerging as an important contributor to exacerbated autoimmunity in MG patients and experimental autoimmune MG animals. Th17 cells and IL-17, as B-cell helpers, can not only directly affect the survival, proliferation, and differentiation of human B cells but also trigger antibody production (40). In anti-AChR antibody-positive MG patients, the Th17 cells frequency and IL-17 level were increased and positively correlated with antibody titer and disease severity (41–43). In this study, ofatumumab significantly decreased the Th17 cells frequency and IL-17 level. Furthermore, ofatumumab also affected the frequency and reactivity of Th17 cells in patients with MS (44, 45).

IL-6 is a pleiotropic inflammatory cytokine. As a regulator of B cells and T cells function, IL-6 promotes T cells activation and B cells proliferation and differentiation (7). Moreover, IL-6 can promote Tfh cells and Th17 cells differentiation (46). The serum level of IL-6 was increased in patients with anti-AChR antibody-positive MG and was correlated with the severity of MG (47). In this study, ofatumumab decreased the IL-6 level in MG patients, which suggests that the immunoregulatory effect of ofatumumab in MG might also involve reducing the IL-6 level.

MG is an autoimmune disease mediated by B cells. Autoantibodies produced by B cells or PCs are thought to be closely related to the pathogenesis of MG (14). In anti-AChR antibody-positive MG patients, the increases in B cells frequency and antibody titers were associated with the exacerbation of MG (48). In this study, B-cell depletion was observed after treatment with ofatumumab; however, no significant change in antibody levels was observed. Ofatumumab has been reported to successfully deplete B cells in the treatment of MS (49, 50). The majority of circulating immunoglobulin was secreted by long-lived PCs lacking CD20 on their surface, including anti-AChR autoantibodies. Specific B cells (CD20+) producing anti-AChR autoantibodies could also be found in the circulation, and their levels were often positively associated with serum autoantibody titers (51). Due to the contribution from AChR-specific B cells sensitive to B-cell depletion and long-lived PCs differing in each patient (52), the change in anti-AChR antibody titers was also variable after B-cell depletion by ofatumumab treatment in patients. The anti-AChR antibody titer showed no significant decrease after statistical analysis, which suggested that the autoreactive PC pool might be more entrenched. In addition, when targeting CD20, the anti-AChR antibody levels did not change notably after treatment with rituximab, while the clinical symptoms were still improved (29). After rituximab treatment, most patients with MG did not show a significant reduction in the anti-AChR antibody level, which was independent of clinical improvement (53); this data supports our results. Accumulated evidence suggests that B-cell depletion therapy has a regulatory effect on T cell subsets. The immunomodulatory effect of rituximab on T cells in patients with MG has been repeatedly reported (30, 54). Furthermore, rituximab or ofatumumab also plays an immunomodulatory role on T cells in other autoimmune diseases, such as MS and rheumatoid arthritis (44, 55, 56). However, the mechanism by which B-cell depletion exerts a regulatory effect on T cells is unknown. The study found that B cells are necessary for the survival of Tfh cells (57). In response to self-antigens, B cells can stimulate the proliferation of Th17 cells and the production of IL-17 by Th17 cells (58). Therefore, the diminished effect of B cells on T cells after B-cell depletion may contribute to the change in T-cell subsets.

Although our study achieved exciting results, only 31 patients were included, and the observation period of 3 months was an impressive shortcoming. Furthermore, non-blinding or other immunosuppressive agents may have affected our results to some extent. For the first time, we have identified the clinical efficacy and partial immunomodulatory mechanism of ofatumumab in patients with MG. This motivates us to observe the long-term efficacy of ofatumumab on MG in a larger cohort of subjects, which may reveal additional effects and reduce the risk of type I statistical errors. This study provides support for conducting randomized controlled prospective studies and animal experiments to further determine the effect of ofatumumab in MG.

## 5 Conclusion

A significant reduction in the QMG, MG-QOL15, and MG-ADL scores was observed in anti-AChR antibody-positive MG patients after one cycle of treatment with ofatumumab; these decreased scores were maintained for a further 2 months. The immunomodulatory effects of ofatumumab may involve the depletion of B cells, reduction in Tfh and Th17 cells frequencies, and reduced IL-6, IL-21, and IL-17 levels during the observation period of 3 months.

## Data availability statement

The raw data supporting the conclusions of this article will be made available by the authors, without undue reservation.

## Ethics statement

The studies involving humans were approved by Ethics Committees of Peking University People's Hospital. The studies were conducted in accordance with the local legislation and institutional requirements. The participants provided their written informed consent to participate in this study. Written informed consent was obtained from the individual(s) for the publication of any potentially identifiable images or data included in this article.

## Author contributions

SL: Writing—original draft. ZZ: Data curation, Writing—original draft. ZL: Project administration, Writing—review & editing.
